# Chemical Profile and Antioxidant Activity of the Oil from Peony Seeds (*Paeonia suffruticosa* Andr.)

**DOI:** 10.1155/2017/9164905

**Published:** 2017-09-26

**Authors:** Xin Yang, Di Zhang, Li-min Song, Qian Xu, Hong Li, Hui Xu

**Affiliations:** ^1^School of Chemistry and Chemical Engineering, Yantai University, Yantai 264005, China; ^2^School of Pharmacy, Yantai University, Yantai 264005, China; ^3^Key Laboratory of Molecular Pharmacology and Drug Evaluation, Yantai University, Yantai 264005, China; ^4^Collaborative Innovation Center of Advanced Drug Delivery System and Biotech Drugs in Universities of Shandong, Ministry of Education, Yantai 264005, China

## Abstract

Peony seed oil (PSO) is a novel vegetable oil developed from the seeds of *Paeonia suffruticosa* Andr. The present study aimed to make an overall investigation on the chemical profile and antioxidant activities of PSO for reasonable development and utilization of this new resource food. Chemical analysis revealed that PSO was characterized by an uncommon high portion of *α*-linolenic acid (>38%), fairly low ratio of n-6 to n-3 polyunsaturated fatty acids (0.69), and much higher content of *γ*-tocopherol than various conventional seed oils. *In vitro* assay indicated that PSO is a more potent scavenger of free radicals than extra virgin olive oil. Moderate intake of PSO exhibited obvious protection against various oxidative damages such as tetrachloromethane-induced acute liver injury in mice and diet-induced hyperlipidemia in rats. The changes in the key indicators of oxidative injury and fatty acid composition in the liver caused by PSO administration were measured, and the results demonstrated that antioxidant properties of PSO are closely related to their characteristic chemical composition. Consequently, the present study provided new evidence for the health implications of PSO, which deserves further development for medical and nutritional use against oxidative damages that are associated with various diseases.

## 1. Introduction

Herbaceous peony (*Paeonia suffruticosa* Andr.) is a kind of traditional Chinese ornamental plant widely cultivated in China, America, Europe, and other Asian areas. In addition to ornamental utilization for their attractive flowers, most of peony species have been also used as medicinal plants, and the studies have mainly focused on paeonol, paeoniflorin, and other bioactive components in corolla, leaf, and root bark for a long time [1–3]. The dry root bark of peony, named mudanpi in Chinese, has been officially recorded in all editions of Chinese pharmacopoeia (ChP) and widely used in compound traditional Chinese medicine preparations for promoting blood circulation and removing blood stasis. Peony seeds are the main by-product of mudanpi with an average annual yield up to 3750 kg per hectare. However, almost all the peony seeds have been just taken as industrial waste. During the newly past decade, this plant resource is attracting great interests for further development since peony seeds, especially the seeds' oil, have been found rich in unsaturated fatty acids, amino acids, stilbenoids, and other nutrient substances [4, 5].

A variety of methods has been applied to extract peony seed oil (PSO), which includes expeller pressing, organic solvent extraction, microwave-assisted extraction, ultrasonic-assisted extraction, and supercritical carbon dioxide-assisted extraction [6–9]. A maximum PSO yield could be up to 24%–27% by using the supercritical CO_2_ extraction method under optimal conditions, and even the traditional expeller pressing method could provide a PSO yield up to 20% [10, 11]. Chemically, PSO is a precious vegetable oil resource rich in unsaturated fatty acids (UFAs, >80%) and characterized with a high proportion of n-3 fatty acids. It has been reported that the majority of fatty acids in PSO are *α*-linolenic acid (ALA) and linoleic acid, and the content of ALA could even be up to 33.8–67.1%, a very uncommon level of n-3 UFAs in plant-derived oils with the exception of flaxseed and perilla seed oil [11]. Due to the high level of ALA, it was approved as a new resource food by the Ministry of Health of China in 2011 [12]. Studies have also shown versatile bioactivities of PSO, such as antibacterial, antidiabetic, hypolipidemic, and sun-screening effects. The safety of PSO has been demonstrated by a series of experiments, including acute toxicity, subacute toxicity, long-term toxicity, and sperm abnormal genetic toxicity [13]. All these findings indicate potential benefits of PSO as a new resource food for dietary supplementation.

It has been well demonstrated that reactive oxygen species (ROS), the products of NADPH oxidase during oxidative phosphorylation, are normal components of healthy cells and are widely involved in phagocytosis, apoptosis, and detoxification as mediators of the first defensive actions of cells. Meanwhile, ROS are bound up with various tissue damages due to the mutagenic effect on all types of cells via the oxidation of DNA, membrane lipids, and proteins [14]. A large number of evidences have also indicated that the health benefits of natural products are closely associated with antioxidant activities through reducing oxidative phenomena and lowering ROS-related diseases and damage, and researches have focused on the discovery and development of safe and effective antioxidants from natural sources [15]. However, there are few reports on systematic investigation of antioxidant activity of PSO *in vitro* and *in vivo*, especially those combining with research on its chemical composition. The main purpose of the present study thus was to investigate antioxidant activities of PSO through various *in vitro* assays and *in vivo* models. The free radical-scavenging activities *in vitro* were evaluated using both exogenous and endogenous systems, and its protective effects against oxidative damages in living organisms induced by tetrachloromethane (CCl_4_) or a high-fat diet were explored. Meanwhile, the overall chemical profile of PSO and the changes in fatty acid composition in mouse liver caused by PSO administration were analyzed to expound the main constituents responsible for its antioxidant capacity. The findings would provide new evidence for reasonable development and utilization of this new resource food.

## 2. Materials and Methods

### 2.1. Materials and Chemicals

The test sample of PSO was a product from the Heze Ruipu Peony Technology Development Corporation (Shandong, China) prepared by a traditional cold pressing method. Extra virgin olive oil (EVOO, Olivoila®, Italy) used as control was purchased from a local supermarket. For both oil samples, the main chemical indices including acid value, iodine value, peroxide value, and saponification value were detected to measure up to Chinese national standard requirements for edible vegetable oil. Gallic acid, *α*-tocopherol, 1,1-diphenyl-2-picrylhydrazyl (DPPH), Folin-Ciocalteu reagent, carboxymethylcellulose (CMC) sodium, and bovine serum albumin (BSA) were obtained from Sigma-Aldrich Co. (St. Louis, MO, USA). Salvianolic acid A (SAA), a kind of naturally occurring polyphenolic compound with potent antioxidant property [16], was kindly provided by Shandong Target Drug Co. Ltd. (Yantai, China), and Xuezhikang capsule was the product of Beijing Peking University WBL Biological Technology Co. Ltd., China. All other chemicals were of the highest grade available.

### 2.2. Assay of Fatty Acids and Unsaponifiable Matters

PSO (5 g) was hydrolyzed with 20 mL of 2 M KOH in methanol at 60°C for 30 min. The unsaponifiable fraction was extracted using the method of Wang et al. [17]; meanwhile, the saponifiable fraction was converted to fatty acid methyl esters (FAMEs) and extracted into hexane according to the method of Zhou et al. [9]. For both fractions, the residues were dried under vacuum and weighed after the extraction solvents were removed. The percentage weight of unsaponifiable matters (*R*_um_) and fatty acids (*R*_fa_) in PSO was calculated as *W*_uf_/*W*_oil_ × 100% and (1 − *W*_uf_/*W*_*oil*_) × 100%, respectively, where *W*_uf_ meant the weight of unsaponifiable fraction and *W*_oil_ the weight of PSO. Then both residues were redissolved in hexane and subjected to analysis by gas chromatography-mass spectrometry (GC-MS) using a Shimadzu QP2010 Plus GC-MS system (Kyoto, Japan) according to conditions previously reported [9, 17]. Qualitative analysis was conducted by matching peak mass fragmentation patterns with those in NIST05 mass spectrum libraries, and quantitative analysis was carried out by normalizing peak areas to obtain the percentage content of each component, *P*_ep_, from which its rate in PSO (*R*) could be calculated by multiplying with *R*_um_ or *R*_fa_.

### 2.3. Assay of Total Tocopherols and Total Phenolics

After extraction by the method of Li et al. [18], total tocopherols in PSO were assayed using a Hitachi F7000 fluorescence spectrophotometer (Tokyo, Japan) with *α*-tocopherol as reference. The 280 nm wavelength was used for excitation, and 324 nm for emission. Total phenolics in PSO were extracted with methanol and then determined using the Folin-Ciocalteu reagent according to the method of Xie et al. [19]. Gallic acid was used as reference standard, and the results were expressed as gallic acid equivalents (GAE) in PSO.

### 2.4. Determination of Antioxidant Activities *In Vitro*

#### 2.4.1. DPPH Scavenging Assay

DPPH is a kind of stable free radical with a strong absorption band centered at about 520 nm, leading to a deep violet color of DPPH radical in solution. It will become colorless or pale yellow when neutralized, which allows for monitoring radical concentration to evaluate radical-scavenging activity from the change in optical absorption [20]. The scavenging activity with respect to free radical DPPH was determined by a method described by Wang et al. [21] with slight modifications. In brief, 4.6 mL aliquots of test samples dissolved in DMSO at concentrations of 0–50 mg·mL^−1^ were mixed with 0.4 mL fresh ethanolic DPPH solution (1 mM) and then let stand at room temperature for 30 min. At the same time, the mixture without test sample was prepared as blank control. Then the absorbance was measured at 517 nm using a Shimadzu UV2550 spectrophotometer (Kyoto, Japan). The percentage DPPH scavenging activity expressed as %scavenging was calculated by the equation (1 − *A*_A_/*A*_B_) × 100%, where *A*_A_ and *A*_B_ were the absorbance values of the test sample and blank, respectively.

#### 2.4.2. Hydroxyl Radical Scavenging Assay

Using the Fenton reaction model system, the assay of hydroxyl radical scavenging activity was conducted according to the method of Lin [22] with some modifications. Hydroxyl radical scavengers have the ability to quench hydroxyl radicals, which could be trapped by salicylic acid and lead to producing dihydroxybenzoic acids that have a characteristic absorption at 510 nm. The hydroxyl radicals were generated by the reaction of Fe (II) complex with H_2_O_2_, and the scavenging activity was determined as follows. Briefly, 2.0 mL aliquot of test sample dissolved in ethanol was mixed with 0.6 mL aqueous solution of FeSO_4_ (8 mM) and 2.0 mL salicylic acid solution in ethanol (3.0 mM). After 0.5 mL H_2_O_2_ (0.6%, *v*/*v*) was added to start the reaction, the mixture was immediately subjected to incubation at 37°C for 30 min, then cooled down to room temperature, and centrifuged at 2000 rpm for 10 min to yield supernatant for absorbance measurement at 510 nm using a Shimadzu UV2550 spectrophotometer (Kyoto, Japan). Meanwhile, the control was made up from 0.5 mL distilled water instead of H_2_O_2_, and the mixture with 2.0 mL ethanol in place of the test sample was prepared as blank. The percentage inhibitor activity expressed as %scavenging was calculated by [1 − (*A*_A_ − *A*_C_)/*A*_B_] × 100%, where *A*_A_, *A*_B_, and *A*_C_ referred to the absorbance value of the test sample, blank, and control, respectively.

For both models, EVOO and *α*-tocopherol were used as positive controls, and the concentration for 50% of maximal scavenging effect, EC_50_, was calculated for quantitative comparison.

### 2.5. Determination of Antioxidant Activities *In Vivo*

#### 2.5.1. Experimentation on Animals

The antioxidant activities *in vivo* were investigated using the mouse model of acute hepatic injury induced by CCl_4_ and hyperlipidemia rats induced by a high-fat diet according to the methods previously reported with some modifications [15, 23]. The animals, including healthy adult male Kunming mice and Sprague-Dawley (SD) rats, were supplied by the Experimental Animal Center of Shandong Luye Pharmaceutical Co. Ltd. (Yantai, China), housed under a 12-hour light/dark cycle in an approved animal house facility maintained at 22 ± 2°C and 40% to 60% relative humidity, given ad libitum access to food and water, and allowed to acclimate for one week prior to experimentation. All of the animal protocols and experiments were approved by the Committee for Ethics of Animal Experiments of Yantai University and complied with National Institutes of Health Guides for Care and Use of Laboratory Animals.

Eighty mice weighing 20 ± 2 g were randomly allocated into six groups for a consecutive 4-week experiment, including the normal control group (NC, *n* = 8), model control group (MC, *n* = 8), positive control group (MC + P, *n* = 16), and the low, mid, and high doses of PSO-treated groups (MC + L, MC + M, and MC + H; *n* = 16 for each group). The mice in the MC + P, MC + L, MC + M, and MC + H groups were administered once daily by oral gavage with SAA (7.5 g·kg^−1^ d^−1^) and PSO (1.3, 4.0, or 12.0 g·kg^−1^ d^−1^) suspended in 1% Na-CMC, and those in the NC and MC groups with equivalent volume of suspending agent, respectively. One day after the last administration, all the mice except those in the NC group were intraperitoneally injected with CCl_4_ (0.125% in peanut oil, *v*/*v*) at a dosage of 10 mL·kg^−1^ to produce acute liver injury, whereas the NC group was given equivalent volume of peanut oil.

Male Sprague-Dawley rats weighing 200 ± 20 g were randomly assigned to six groups (*n* = 6) for a consecutive 30-day experiment, including the normal diet group (ND), high-fat diet model group (HFD), positive control group (HFD + P), and the low, mid, and high doses of PSO-treated groups (HFD + H, HFD + M, and HFD + L). The animals in the ND group were daily fed with a normal standard laboratory diet composed in accordance to GB14924.3-2010 (Shanghai Keaoxieli Feed Co. Ltd., China), while the other five groups were fed with a commercial AIN-76 rodent diet (Seebio Biotech (Shanghai) Co. Ltd., China) for the hyperlipidemia model. Meanwhile, the rats in the HFD + P, HFD + L, HFD + M, and HFD + H groups were intragastrically administered once daily with Xuezhikang (120 mg·kg^−1^ d^−1^) and PSO (1.0, 2.5 or 6.0 g·kg^−1^ d^−1^) suspended in 1% Na-CMC, and those in ND and HFD groups with equivalent volume of suspending agent, respectively. The dosages were based on the previously reported methods and the recommended human intake of PSO [12, 23].

For both models, body weight was recorded every 2 days, and the animals were fasted overnight and sacrificed at the end of the experimental period. The liver was quickly removed, rinsed thoroughly with ice-cold saline, blotted, and weighed. Serum was obtained by centrifugation (4°C, 3000 rpm × 10 min). All serum and liver samples were stored at −80°C until analysis.

#### 2.5.2. Biochemical Analysis

Liver homogenates were prepared with ice-cold saline (10%, *w*/*v*) for biochemical analysis. The oxidative stress indices including malondialdehyde (MDA) content and activity of superoxide dismutase (SOD), glutathione peroxidase (GPX), aspartate aminotransferase (AST), and alanine aminotransferase (ALT) in the serum or liver were detected to evaluate antioxidant activity using commercial kits (Nanjing Jiancheng Bioengineering Institute, China). The lipid levels including serum total cholesterol (TC), triglyceride (TG), low-density lipoprotein cholesterol (LDL-C), and high-density lipoprotein cholesterol (HDL-C) were detected to evaluate hyperlipidemic activity in rats using commercial kits (Shanghai Zhicheng Biological Technology Co. Ltd., China) according to the manufacturer's instructions. Contents of protein in the serum or liver were determined by the Bradford method using BSA as a standard.

#### 2.5.3. Analysis of Fatty Acid Constituents in Mouse Liver

For the mice in the MC group and PSO-treated groups (MC + L, MC + M, and MC + H), one-half of the liver samples were homogenate for constituent analysis of fatty acid. Lipids were extracted by diethyl ether, and the supernatants from a triple extraction were combined and evaporated in vacuo. The residue was weighed to determine extraction yield of lipids (*Y*_e_, mg/g liver) and then subjected to saponification and methyl esterification to obtain FAMEs. The analysis of FAMEs was carried out on a Shimadzu QP2010 Plus GC-MS (Kyoto, Japan) equipped with a RTX5 capillary column (30 m × 0.25 mm i.d., 0.25 mm film thickness). The optimal assay conditions were as follows. The oven temperature was initially held at 150°C for 2 min, then raised to 230°C at the rate of 5°C·min^−1^, and held for 30 min. Injection temperature, interface temperature, and EI ion source temperature were set at 230°C, 230°C, and 200°C, respectively. Helium was used as carrier gas at a flow rate of 0.88 mL·min^−1^. The injection volume was 1 *μ*L, and the split ratio was 1 : 30. The MS was operated in scan (*m/z* 40–500) and SIM (selected ion monitoring) mode. Qualitative identification of FAMEs was conducted by matching mass fragmentation patterns with those in the NIST05 mass spectrum libraries. The percentage content of each fatty acid (*P*_fa_) was obtained by the peak area normalization method. Then, the content of fatty acid in mouse liver (mg/g liver) could be calculated by multiplying *P*_fa_ by *Y*_e_.

### 2.6. Statistical Analysis

All values were expressed as means ± standard deviation (SD). The one-way analysis of variance (ANOVA) and Tukey's multiple comparison posttest were used for data analysis. Differences less than 0.05 (*P* < 0.05) were considered statistically significant.

## 3. Results and Discussion

### 3.1. Chemical Profile of PSO

The chemical profile of PSO was investigated firstly, and the results of main fatty acids and other nutrient components are shown in Table 1. Fatty acids accounted for 98.46% of the total weight of PSO and mainly consisted of unsaturated fatty acids (UFAs) with the weight percentage up to 89.34%. Although the content and composition of UFAs in plant seed oils may vary with the extraction techniques, PSO is characterized by the dominant abundance in UFAs according to the comparison between the data for present PSO test sample prepared by traditional cold-pressed method and those by supercritical carbon dioxide extraction or solvent extraction method [10]. Moreover, the predominant UFAs in PSO were determined as polyunsaturated fatty acids (PUFAs), including n-3 ALA (38.86%), n-6 linoleic acid (LA, 26.74%), and oleic acid (23.74%), suggesting a high portion of n-3 PUFAs (ALA, more than 38%) and low ratio of n-6/n-3 (0.69) in PSO. It was also coincident with the report of fatty acids in sixty tree peony cultivars that indicated that the ratio of n-6/n-3 was between 0.4 and 1.6 and the ratio of n-3 to total FAs was greater than 38% [24].

Many findings have demonstrated that the composition of polyunsaturated fatty acids in cell membranes is determined largely by diets and both n-3 and n-6 PUFAs are essential fatty acids that need to be supplemented through food. However, n-3 and n-6 PUFAs have different physiological functions and cannot convert mutually in mammalian cells. The diet with a lower n-6/n-3 fatty acid ratio is associated with the lower incidence of cardiovascular disease and longer life expectancy, and balancing the n-6/n-3 PUFAs ratio of dietary supplement has attracted wide attention. The recommended values are generally lower than 10 : 1 according to reports from authority organizations such as FAO, ISSFAL, and ECSCF [25, 26]. Unfortunately, vegetable oils are usually rich in n-6 fatty acids but very low in n-3 fatty acids, then have a very high n-6/n-3 fatty acid ratio, leading to daily intake of n-3 PUFA falls far short of the recommended dosage for human health [27]. With the exception of perilla seed and flaxseed oil, such fatty acid constitutions with high level of ALA and low level of the n-6/n-3 fatty acid ratio as those detected for PSO are uncommon in seed oils. Taking into account that ALA is the precursor of eicosapentaenoic acid (EPA) and docosahexaenoic acid (DHA), the most important long-chain n-3 PUFAs playing key roles in various physiological functions, PSO thus could be a promising ALA source for dietary supplement of n-3 fatty acids.

The content of tocopherols is another important criterion for the assessment of seed oils since they are fat-soluble antioxidants protecting PUFAs from lipid peroxidation and regulating free radical production in human body for the prevention and treatment of many diseases [28]. As shown in Table 1, the content of total tocopherols in PSO was determined as 76.0 ± 3.1 mg·100 g^−1^, which was much higher than that of common vegetable oils from *Juglans regia* (24.1 mg·100 g^−1^), *Corylus heterophylla* (31.0 mg·100 g^−1^), *Bertholletia excelsa* (31.9 mg·100 g^−1^), and other plants [29]. Meanwhile, *γ*-tocopherol determined by GC-MS showed an average of 68.3 mg·100 g^−1^ and accounted for nearly 90% of the total tocopherol content, indicating that *γ*-tocopherol is present in PSO at significantly higher levels than other tocopherol components. Many recent studies have demonstrated that *γ*-tocopherol may have more potent antioxidant activities than *α*-tocopherol although traditional supplementation primarily focused on *α*-tocopherol [30]. In contrast to EVOO that is abundant in total phenolics (73–265 *μ*g·g^−1^ of gallic acid equivalent) and *α*-tocopherol (25.1–36.9 mg·100 g^−1^) [31], PSO is obviously characterized by its high and predominant content of *γ*-tocopherol. These findings were in line with the results previously reported [32] and revealed that PSO is a good performance nutrient source rich in both n-3 PUFAs and *γ*-tocopherol. Such special chemical profile may also endow PSO with specific benefits such as antioxidant activities to human health.

### 3.2. Antioxidant Activities *In Vitro*

**Figure 1:**
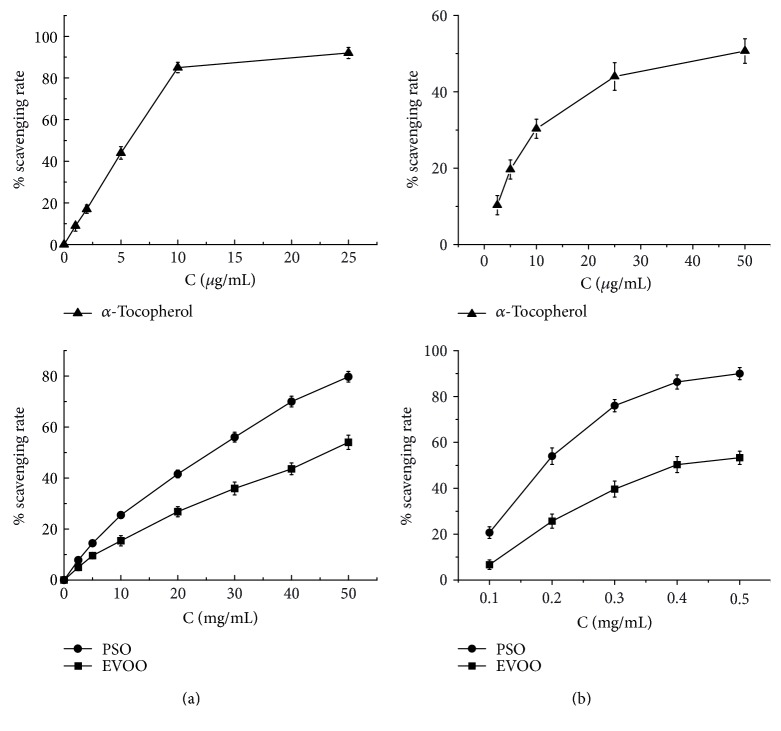
Scavenging activities of PSO in vitro against DPPH (a) and hydroxyl (b) radicals. EVOO and *α*-tocopherol were used as controls, and data were presented mean ± SD of triplicate determinations of the percentage scavenging of free radicals.

DPPH is a well-known trap for other radicals, and *in vitro* DPPH assay is considered a valid accurate, repeatable, and economic method widely used to assess antiradical or antioxidant activity of natural compounds in foods or biological systems [33]. The scavenging activity of PSO against DPPH radicals was firstly examined to evaluate its antioxidant property *in vitro*. As shown in Figure 1(a), both positive controls displayed a concentration-dependent scavenging effect against DPPH radicals. The EC_50_ values were calculated as 5.72 *μ*g·mL^−1^ and 44.92 mg·mL^−1^ for *α*-tocopherol and EVOO, respectively, which were consistent with those previously reported [34]. Meanwhile, the percentage scavenging of DPPH radicals by PSO was measured as 7.8%–79.7% with concentration ranging from 2.5 to 50 mg·mL^−1^. The EC_50_ value was determined as 29.30 mg·mL^−1^ and indicated that PSO is a more potent scavenger of DPPH free radicals when compared with EVOO. Taking into consideration the above-mentioned results of chemical profiling analysis, it is plausible that such difference in antiradical activity between the two seed oils may be closely related to the different chemical constitutions, especially the abundance of n-3 PUFAs and *γ*-tocopherol in PSO. Further, taking into account the fact that PSO contained total tocopherols at an average level of 76.0 mg·100 g^−1^, the EC_50_ of 29.30 mg·mL^−1^ PSO would be equivalent to 22.27 *μ*g·mL^−1^ of tocopherols against DPPH radicals, which is somewhat higher than that of *α*-tocopherol in DPPH assay (5.72 *μ*g·mL^−1^). The interference from those components coexisting with tocopherols in PSO may be responsible for such discrepancy. It has been found that DPPH can only be soluble in organic solvent and the interference of absorbance from sample matrices could lead to widely varying results of antioxidant activity [35]. The influence of organic acids on DPPH· scavenging by ascorbic acid has also been previously reported [36]. With respect to the present PSO sample, an average level of total acid value was determined as 186.6 mg·100 g^−1^, suggesting a certain amount of free fatty acids in PSO, which have the potential to form strong intermolecular interaction by hydrogen bonding with those components containing hydroxyl such as tocopherols and phenolics, then exert influence on estimation of DPPH· scavenging activity.

In addition to the assay of scavenging activity against DPPH·, the antioxidant property of PSO *in vitro* was further evaluated using a model of endogenous free radicals since DPPH radicals are foreign to biological systems, and DPPH assay has limited, if any, relevance to biological systems [37, 38]. The hydroxyl radical is known as the most reactive radical that can easily cross cell membranes and readily react with vital biomolecules such as carbohydrates, proteins, lipids, and DNA in living cells, causing tissue damage or cell death. It has been found that hydroxyl radicals generated close to membranes could attack the fatty acid side chains of the membrane phospholipids and resultantly stimulate lipid peroxidation [39]. Thus, the scavenging activity of PSO against hydroxyl radicals was investigated in the present study by the Fenton reaction. As illustrated in Figure 1(b), both positive controls and PSO exhibited hydroxyl radical scavenging activity in a concentration-dependent manner. The EC_50_ values were calculated as 0.404 mg·mL^−1^ and 37.3 *μ*g·mL^−1^ for EVOO and *α*-tocopherol, respectively. Meanwhile, the percentage scavenging of hydroxyl radicals by PSO was measured as 18–92% with concentration ranging from 0.1 to 0.5 mg·mL^−1^, and the EC_50_ was determined as 0.185 mg·mL^−1^, indicating that PSO is also a more potent scavenger of hydroxyl radicals in contrast to EVOO. When comparing the scavenging potency against the two different free radicals, the EC_50_ values of PSO differed by more than two orders of magnitude (0.185 mg·mL^−1^ for OH· versus 29.30 mg·mL^−1^ for DPPH·), whereas *α*-tocopherol showed much less difference in an opposite direction (37.3 *μ*g·mL^−1^ for OH· versus 5.72 *μ*g·mL^−1^ for DPPH·). Along with the results of DPPH assay, these findings further revealed that PSO acted as a more powerful radical scavenger of hydroxyl radicals, and the constituents mainly responsible for its antiradical activity may be those substances other than *α*-tocopherol in PSO.

### 3.3. Protective Effects against CCl_4_-Induced Oxidative Damages in Mice

Many evidences suggest that intake of antioxidant nutrients from food sources offers health advantages. However, there is a great difference between antiradical and antioxidant activity and they do not necessarily coincide. According to Tirzitis and Bartosz, antiradical activity may characterize the ability of compounds to react with free radicals in a single free radical reaction, whereas antioxidant activity represents the ability to inhibit the process of oxidation, which usually involves a set of different reactions [40]. Consequently, all test systems using a stable free radical give information on the radical scavenging or antiradical activity, and in many cases, it does not correspond to the antioxidant activity. Therefore, *in vivo* studies were further carried out to understand the real antioxidant property of PSO in the present study.

**Figure 2:**
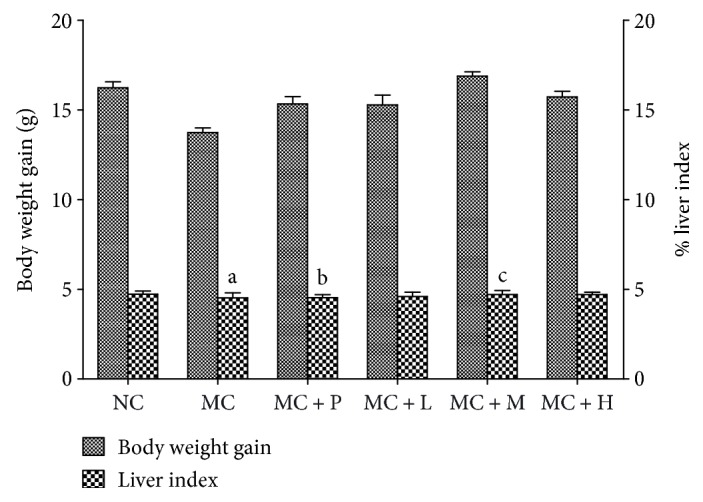
Body weight gain and liver index of mice for a consecutive 4-week experiment. Values expressed as the mean ± SD of 6 mice in each group. ^a^*P* < 0.05, liver index compared with the NC group. ^b^*P* < 0.01, liver index compared with the MC group. ^c^*P* < 0.05, liver index compared with the MC group.

**Figure 3:**
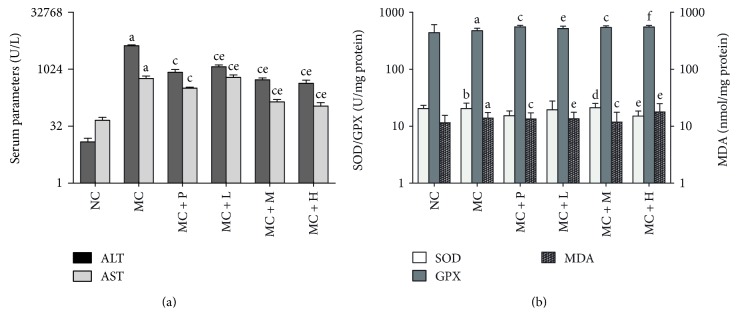
Levels of several serum and liver biological indexes of mice for a consecutive 4-week experiment. Values are expressed as the mean ± SD of 6 mice in each group. ^a^*P* < 0.01, compared with the NC group. ^b^*P* < 0.05, compared with the NC group. ^c^*P* < 0.01, compared with the MC group. ^d^*P* < 0.05, compared with the MC group. ^e^*P* < 0.01, compared with the MC + P group. ^f^*P* < 0.05, compared with the MC + P group.

Firstly, experimental CCl_4_-induced acute hepatic injury in mice was applied to investigate *in vivo* antioxidant activity of PSO as a dietary supplement. It has been demonstrated that the main cause of acute liver injury by CCl_4_ is free radicals (·CCl_3_ or CCl_3_O·) metabolized by the mixed function cytochrome p450 in endoplasmic reticulum of liver cells, which immediately propagate a chain of lipid peroxidation events, give rise to the breakdown of membrane structure, and in end lead to liver cell swelling and inflammatory infiltrates [41]. Resultantly hepatic cell marker enzymes, mainly ALT and AST, would leak into the bloodstream, causing increased levels in the serum [42]. Significant increase in MDA content in the liver could also be observed because of its reaction during the final stages of lipid peroxidation, whereas hepatic levels of SOD and GPX decrease markedly since they are the major antioxidant enzymes responsible for eliminating ROS derived from redox process in the liver, where O_2_^−^· is converted into H_2_O_2_ by SOD and metabolized into nontoxic products by GPX [43]. As shown in Figure 2, there was no significant difference in average body weight gain among all the six groups (*P* > 0.05), suggesting that intake of PSO at a dosage of 1.3 to 12.0 g·kg^−1^·d^−1^ had no adverse effects on mouse growth. As to liver index that is commonly used for the estimation of hepatic lesions and measured as the weight ratio of the liver to the body, the MC group had a much higher value (*P* < 0.05) than the NC group, while significant decrease could be observed in both the MC + P (*P* < 0.01) and the MC + M groups (*P* < 0.05) in contrast to the MC group. With respect to those indicators of chemically induced oxidative damages to hepatic cell membrane and mitochondria, the MC group displayed significant decrease in hepatic levels of GPX (*P* < 0.01) and SOD (*P* < 0.05), but increase in MDA content in the liver and ALT and AST in the serum (*P* < 0.01) when compared with the NC group, obviously indicating the acute liver damage in mice caused by a single intraperitoneal dose of CCl_4_ (Figures 3(a) and 3(b)). Whereas, much lesser changes were observed in those groups preadministered with a positive drug or PSO in contrast to the MC group, further demonstrating effectiveness of such pretreatments. In addition, the MC + M group exerted much higher levels of SOD (*P* < 0.05) and GPX (*P* < 0.01), but lower MDA content in the liver and ALT and AST in the serum (*P* < 0.01) than the MC group did. The comparison between the MC + M and the MC + P groups revealed that effects of PSO at medium dose on hepatic MDA, SOD, and GPX were almost equivalent to those of positive control agent (*P* > 0.05) and even more potent on ALT and AST in the serum (*P* < 0.01). These findings thus indicated that moderate intake of PSO (4 g·kg^−1^·d^−1^) may effectively protect hepatic cells against oxidative damage by mediating ROS generation that would subsequently lead to the stabilization of hepatic cellular membrane and maintenance of normal antioxidant enzyme activity in the liver.

**Figure 4:**
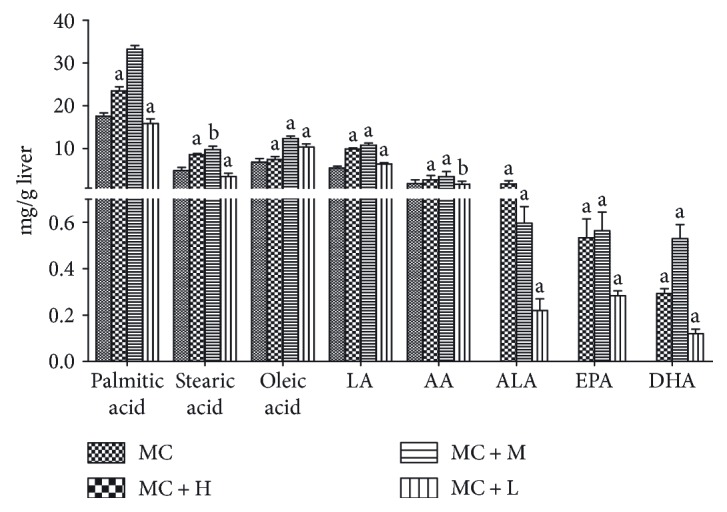
Fatty acids present in mouse livers. Values are expressed as mean ± SD (*n* = 6). ^a^*P* < 0.01, compared with the MC group. ^b^*P* < 0.05, compared with the MC group.

In order to obtain more insight into the major active constituents responsible for the *in vivo* antioxidant effect of PSO, fatty acid constitution in mouse liver was further analyzed by GC-MS. As illustrated in Figure 4, the mice in the MC group and those groups preadministered with PSO (MC + L, MC + M, and MC + H groups) all contained both saturated fatty acids (SFAs) and UFAs in the liver, among which the SFAs were mainly composed of palmitic acid and stearic acid, while the UFAs consisted of oleic acid (18 : 1, n-9), the principal essential PUFAs LA (18 : 2, n-6), ALA (18 : 3, n-3), and their long-chain derivatives arachidonic acid (AA, 20 : 4, n-6), EPA (20 : 5, n-3), and DHA (22 : 6, n-3). Further comparison of such treatment pairs revealed that the mice preadministered with PSO had significantly higher levels of UFAs than the MC group (*P* < 0.05). With respect to contents of n-3 PUFAs, particularly significant difference could be observed since they were all below the limit of detection in the MC group, but obviously present in those groups pretreated with PSO at levels of 0.22–1.12 mg·g^−1^, 0.26–0.57 mg·g^−1^, and 0.10–0.53 mg·g^−1^ for ALA, EPA, and DHA, respectively. Moreover, the MC + M group had much lower contents of SFAs than the MC and MC + H groups and contained the highest contents of EPA and DHA. Such composition of PUFAs in animals pretreated with PSO indicated the conversion of ALA to n-3 long-chain PUFAs also could account for the protective effect of PSO on CCl_4_-induced liver injury discussed above, since they are the most important n-3 PUFAs playing key roles in mediating ROS generation and excretion of lipid peroxidation. A similar modulatory effect of PSO on lipid metabolism in hyperlipidemia rats was previously reported [12]. Therefore, it might be concluded that moderate PSO intake may effectively protect hepatic cells against oxidative damage, which is closely associated with special fatty acid constitution of PSO, such as above-mentioned abundance of ALA and low n-3/n-6 essential fatty acid ratio.

### 3.4. Effects on Lipid Peroxidation in Hyperlipidemia Rats

In recent years, human dietary lipid intake has shifted towards PUFAs, especially n-3 PUFAs for their hypocholesterolemic effects. The hypolipidemic activity of PSO used as a dietary supplement has been lately reported in rats fed with a high-fat diet, and the findings demonstrate that PSO could participate in the regulation of plasma lipid concentration and cholesterol metabolism in the liver through inhibition of lipogenesis and upregulation of fatty acid *β*-oxidation [12]. In the present study, the *in vivo* antioxidant effect of PSO against lipid peroxidation in hyperlipidemia rats was further investigated to achieve more insight into the health-promoting characteristics of such vegetable oil with unique chemical composition.

**Figure 5:**
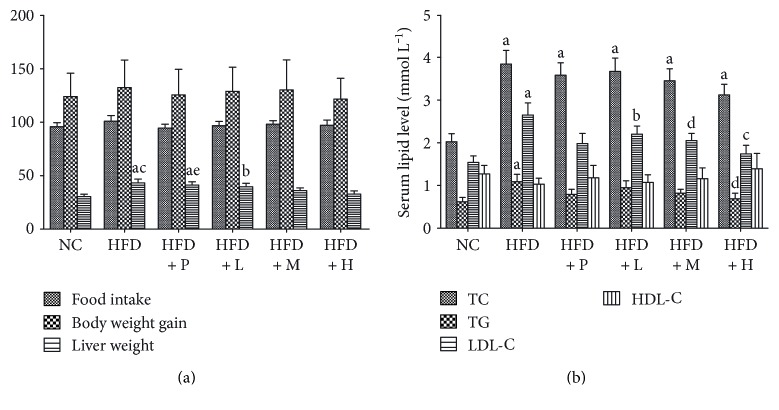
The levels of food intake, body weight gain, liver weight, and serum lipid profile of different feeding groups for a consecutive 30-day experiment including NC (normal diet), HFD (high-fat diet), HFD + P (Xuezhikang, 120 mg kg^−1^ d^−1^), HFD + L (PSO, 1.0 g kg^−1^ d^−1^), HFD + M (PSO, 2.5 g kg^−1^ d^−1^), and HFD + H (PSO, 6.0 g kg^−1^ d^−1^). Data were expressed as mean ± SD (*n* = 6), and means with different letters were different at the *P* < 0.05 level. Different lowercase letters indicated differ significantly (*P* < 0.05). ^a^*P* < 0.01, compared with the NC group. ^b^*P* < 0.05, compared with the NC group. ^c^*P* < 0.01, compared with the HFD group. ^d^*P* < 0.05, compared with the HFD group. ^e^*P* < 0.05, compared with the HFD + P group.

As illustrated in Figure 5(a), there were no significant differences in body weight gain among the six groups. Although the animals in the HFD + H group displayed the least weight gain, the weight-lowering function of PSO was not related to appetite suppression, since insignificant differences in daily food intake were observed. However, the HFD group showed significantly higher liver weight, as well as serum levels of TC, TG, and LDL-C (*P* < 0.01), while lower level of HDL-C in the serum when compared with the NC group (Figure 5(b)). Such differences between the two groups thus indicated a successful diet-induced hyperlipidemia model accompanying with liver damage in rats. In contrast to the HFD group, those groups simultaneously fed with PSO exhibited dose-dependent changes in these parameters, which were in line with the beneficial results of PSO previously reported [12] and also indicated its hypolipidemic potential as a diet supplement due to effective improvement of the atherogenic lipoprotein profile.

**Figure 6:**
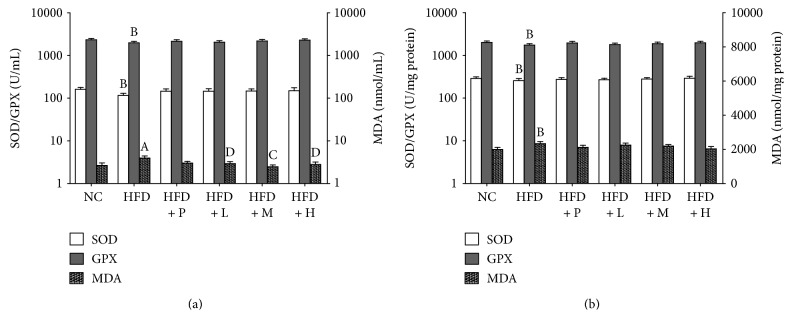
The activities of SOD and GPX and MDA level in the serum (a) and liver (b) of each group for a consecutive 30-day experiment. Data were expressed as mean ± SD (*n* = 6). Different lowercase letters indicated differ significantly (*p* < 0.05). ^A^*P* < 0.01, compared with the NC group. ^B^*P* < 0.05, compared with the NC group. ^C^*P* < 0.01, compared with the HFD group. ^D^*P* < 0.05, compared with the HFD group. SOD: superoxide dismutase; GPX: glutathione peroxidase; MDA: malondialdehyde. NC: normal diet; HFD: high-fat diet; HFD + P: Xuezhikang (0.12 g kg^−1^ d^−1^); HFD + L: PSO (1.0 g kg^−1^ d^−1^); HFD + M: PSO (2.5 g kg^−1^ d^−1^); HFD + H: PSO (6.0 g kg^−1^ d^−1^).

It has been indicated that high-fat diets could induce oxidative stress in a variety of tissues, and liver injury or hepatotoxicity is one of the main relative consequences of hyperlipidemia [44]. Thus, the protective effect of PSO against hepatic damage caused by hyperlipidemia was further evaluated. Several main markers of oxidative stress-induced injury in the serum and liver were measured, which included MDA content, an indicator of lipid peroxidation level, and the activities of SOD and GPX that are common indices of intrinsic antioxidant capacities. As illustrated in Figure 6, SOD and GPX activities decreased significantly and MDA level increased remarkably in the HFD group when compared with those in the NC group (*P* < 0.05). In addition, all PSO treatments improved the levels of these indicators in both the serum and the liver in a dose-dependent manner, and the improving effects of PSO at medium to high dose were even more potent than the positive control agent. Considering the above-mentioned improving effects on lipid metabolism, these observations demonstrated that PSO supplementation does not cause hepatotoxicity but could prevent oxidative damage due to a high-fat diet, suggesting such a hypolipidemic effect that is closely related to antioxidant capacity in terms of PSO.

## 4. Conclusion

In conclusion, the present study was conducted to achieve an overall insight into the chemical profile and antioxidant property of PSO, a new resource food developed from the seeds of peony. For the first time, the findings demonstrated that PSO possesses a potent scavenging effect against free radicals as well as *in vivo* antioxidant capacity by protecting living organs from oxidative damage due to various causes such as an exogenous free radical-generating agent and lipid peroxidation from diet-induced hyperlipidemia. These antioxidant properties of PSO are closely related to the special chemical composition that is characterized by high contents of ALA and *γ*-tocopherol, especially the low ratio of n-6/n-3 PUFAs originating from its uncommon abundance in ALA. Consequently, the antioxidant activities could have contributed, at least partially, to the health implications of PSO, and its antioxidant-related therapeutic benefits deserve further investigation.

## Figures and Tables

**Table 1:** Contents of main fatty acids, unsaponifiable matters, total tocopherols, and phenolics in PSO.

Components	Contents^a^
(A) Main fatty acids (%)	
Palmitic acid	7.5 ± 2.8
Stearic acid	1.8 ± 0.2
Oleic acid	24.1 ± 3.7
LA	27.2 ± 1.7
ALA	39.5 ± 5.1
(B) Unsaponifiable matters (mg/100 g)	
*γ*-Tocopherol	63.4 ± 2.6
Stigmasterol	30.8 ± 1.2
*γ*-Sitosterol	955 ± 33
Fucosterol	248 ± 17
(C) Total tocopherols (mg/100 g)	76.0 ± 3.1
(D) Total phenolics (mg/100 g)	3.34 ± 0.15

^a^Data are expressed as the mean ± SD of three replicates (*n* = 3).
